# Comprehensive bioinformatics analysis identifies LAPTM5 as a potential blood biomarker for hypertensive patients with left ventricular hypertrophy

**DOI:** 10.18632/aging.203894

**Published:** 2022-02-14

**Authors:** Tiegang Li, Weiqi Wang, Wenqiang Gan, Silin Lv, Zifan Zeng, Yufang Hou, Zheng Yan, Rixin Zhang, Min Yang

**Affiliations:** 1 State Key Laboratory of Bioactive Substances and Function of Natural Medicine, Institute of Materia Medica, Chinese Academy of Medical Sciences and Peking Union Medical College, Beijing 100050, China

**Keywords:** left ventricular hypertrophy (LVH), hypertension, LAPTM5, bioinformatics analysis, biomarker

## Abstract

Left ventricular hypertrophy (LVH) is a pivotal manifestation of hypertensive organ damage associated with an increased cardiovascular risk. However, early diagnostic biomarkers for assessing LVH in patients with hypertension (HT) remain indefinite. Here, multiple bioinformatics tools combined with an experimental verification strategy were used to identify blood biomarkers for hypertensive LVH. GSE74144 mRNA expression profiles were downloaded from the Gene Expression Omnibus (GEO) database to screen candidate biomarkers, which were used to perform weighted gene co-expression network analysis (WGCNA) and establish the least absolute shrinkage and selection operator (LASSO) regression model, combined with support vector machine-recursive feature elimination (SVM-RFE) algorithms. Finally, the potential blood biomarkers were verified in an animal model. A total of 142 hub genes in peripheral blood leukocytes were identified between HT with LVH and HT without LVH, which were mainly involved in the ATP metabolic process, oxidative phosphorylation, and mitochondrial structure and function. Notably, lysosomal associated transmembrane protein 5 (LAPTM5) was identified as the potential diagnostic marker of hypertensive LVH, which showed strong correlations with diverse marker sets of reactive oxygen species (ROS) and autophagy. RT-PCR validation of blood samples and cardiac magnetic resonance imaging (CMRI) showed that the expression of LAPTM5 was significantly higher in the HT with LVH model than in normal controls, LAPTM5 demonstrated a positive association with the left ventricle wall thickness as well as electrocardiogram (ECG) parameters widths of the QRS complex and QTc interval. In conclusion, LAPTM5 may be a potential biomarker for the diagnosis of LVH in patients with HT, and it can provide new insights for future studies on the occurrence and the molecular mechanisms of hypertensive LVH.

## INTRODUCTION

Hypertensive left ventricular hypertrophy (LVH) is a well-recognized risk factor for heart failure, myocardial infarction, arrhythmias, sudden cardiac death, and stroke [1, 2]. Therefore, early-stage screening and diagnosis of LVH is important to minimize the impact of cardiovascular events as well as cardiovascular disease (CVD)-related morbidity and mortality.

In clinical practice, LVH can be assessed by electrocardiography (ECG), echocardiography, computed tomography (CT), and cardiac magnetic resonance imaging (CMRI). However, detection of LVH is subject to various limitations, such as low sensitivity and specificity, lengthy diagnostic time, technical complexity, and expenses [3]. This has led to accelerate research on the use of circulating biomarkers as diagnostic and prognostic tools for LVH in patients with hypertension, as it is difficult to obtain tissue biopsies. Circulating biomarkers for hypertensive heart disease, including brain natriuretic peptide (BNP), pro-BNP, soluble ST2 (sST2), C-reactive protein (CRP), cardiotrophin-1, and the ratio of neutrophil-to-lymphocyte [4–7] have been extensively investigated. Despite the high clinical and diagnostic value of these biomarkers, more practical biomarkers are required to enhance the early detection and the diagnostic accuracy of cardiac disease. Therefore, the development of novel biomarkers that reflect hypertensive LVH and complement the existing biomarkers would address a significant unmet clinical need.

With the development of high-throughput microarray and sequencing technology, bioinformatics analysis offers an ideal way to screen large gene expression datasets to study the molecular mechanisms of diseases and discover disease-specific biomarkers [8–10]. A previous study demonstrated an association between ZNF606 gene expression from monocytes and the risk of coronary artery disease (CAD), especially in patients with multiple coronary artery stenosis [11]. Consistently, recent research has shown that the screened core clusters of genes in blood samples may be a target for the diagnosis and treatment of coronary atherosclerotic heart disease (CHD) as a molecular typing module [12]. However, little data has been reported on the bioinformatics-based identification of potential blood biomarkers for predicting LVH in hypertensive patients.

In this study, we firstly evaluated the gene expression profiles in peripheral blood leukocytes from normal individuals and hypertensive patients without LVH or with LVH based on Gene Expression Omnibus (GEO) datasets. Secondly, we identified related hub genes and explored potential biological functions of these candidate biomarkers using bioinformatics analyses. Finally, the predicted biomarkers of hypertensive LVH were verified in an animal model with experimental methods. Our study provides a more effective and comprehensive way to investigate blood biomarkers for distinguishing hypertensive patients with LVH from those without LVH and their potential clinical significance in cardiac hypertrophy.

## RESULTS

### Profiles of the transcriptomic features in leukocytes among all different groups

**Figure 1:**
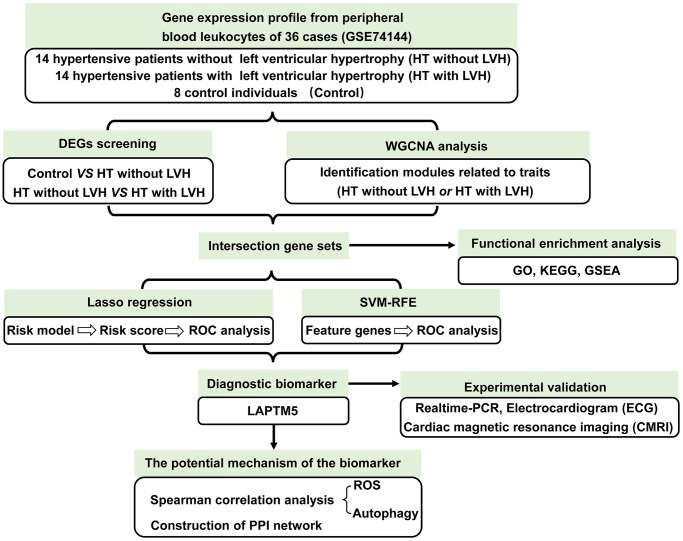
The workflow showing the strategies of the present study.

To identify the feature genes relevant to left ventricular remodeling in hypertensive patients, we analyzed one mRNA expression dataset published in the GEO database (GSE74144), which contained 36 subjects, i.e., eight healthy individuals (Control), 14 hypertensive patients without left ventricular hypertrophy (HT without LVH), and 14 hypertensive patients with left ventricular hypertrophy (HT with LVH). The detailed screening scheme utilized in this study is described in the flow chart (Figure 1).

**Figure 2:**
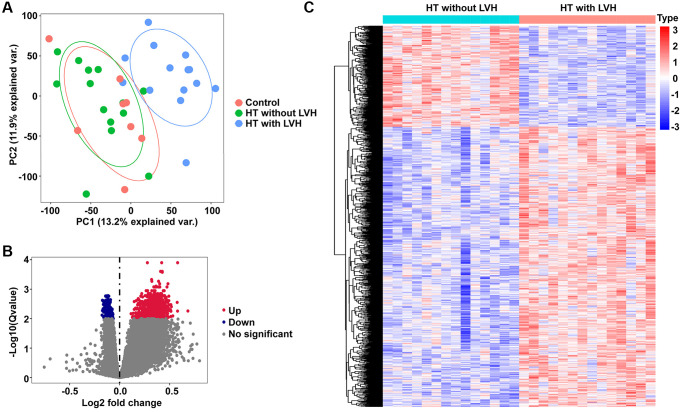
**PCA and DEGs identification.** (**A**) The PCA plots for all the subjects. Red nodes represent the Control sample cluster, green nodes designate the HT without LVH sample cluster, and blue nodes indicate the HT with LVH sample cluster. (**B**) Volcano plots of differential gene profiles between HT with LVH and non-LVH groups. Grey nodes represent genes that are not differentially expressed, red nodes represent significantly upregulated genes, and blue nodes indicate significantly downregulated genes in HT with LVH. (**C**) Heat map of all the DEGs between HT with LVH and non-LVH. Red exhibits overexpression, while blue indicates lower expression of genes in hypertensive LVH.

First, unsupervised principal component analysis (PCA) was applied for all samples prior to identifying the differentially expressed genes (DEGs) so as to inspect the global expression profiling in the dataset. Results of PCA showed that clusters of Control and HT without LVH significantly overlapped, while they displayed completely disjoint populations with HT with LVH (Figure 2A). Then, RNA-seq differential expression analysis was carried out. According to the screening method and criteria discussed above, no DEGs were detected between Control samples and HT without LVH samples, which was consistent with the outcome of the PCA test. In contrast, a total of 1,131 DEGs were obtained in HT with LVH compared with HT without LVH samples, of which 831 were significantly upregulated and 300 were significantly downregulated. Volcano plots were drawn to depict the DEGs of HT with LVH versus HT non-LVH, with red points indicating high expression and blue points representing low expression (Figure 2B). The landscape of DEG expression is displayed in a heat map as well (Figure 2C).

### Construction of co-expression networks and identification of HT with LVH-associated gene co-expression modules

**Figure 3:**
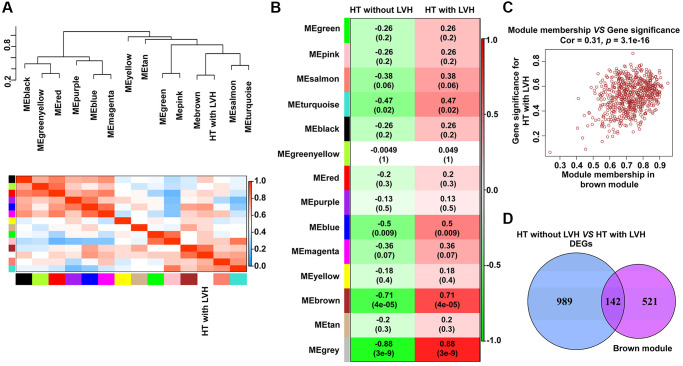
**Identification of modules with clinical traits (HT without LVH or HT with LVH) in WGCNA.** (**A**) The dendrogram shows the relation of modules with HT with LVH and the heat map shows the eigengene adjacency. (**B**) Heat map of the relationship between the module eigengenes and clinical traits (HT without LVH or HT with LVH). Module names are displayed on the left, and each column corresponds to a clinical trait. The number in the first row in each cell represents the Pearson correlation coefficient, and the *p* value of the corresponding module-trait is exhibited in parentheses. The color of each cell indicates the degree of correlation. (**C**) Scatterplot of correlation between MEbrown membership and gene significance for hypertensive LVH. The correlation coefficient and *p* value are listed above the scatterplots. One dot represents one gene in the brown module. (**D**) Venn diagrams showing the number of common genes within brown module and DEGs identified from HT with LVH and non-LVH samples.

A total of 13 distinct gene co-expression modules were generated via WGCNA analysis (Supplementary Figure 1). The relevance between each module and clinical traits (HT without LVH or HT with LVH) were tested. The eigengene dendrogram and heat map indicated that the MEbrown modules highly correlated with hypertensive LVH (Figure 3A). According to the eigengene dendrogram and heatmap plot, the results demonstrated that the brown module displayed a positive relationship with HT with LVH (r = 0.71, *p* = 4e-05) and negative relationship with HT without LVH (r = −0.71, *p* = 4e-05) (Figure 3B). Gene significance and module membership were plotted for the brown module, indicating that this module was significantly related to hypertensive LVH (r = 0.31, *p* = 3.1e-16) (Figure 3C). Finally, 142 genes shared by brown module and DEGs derived from HT without LVH or hypertensive LVH samples were obtained to implement functional enrichment analyses (Figure 3D). Of note, all 142 genes were overexpressed in HT with LVH samples.

### Functional and pathway enrichment analysis

**Figure 4:**
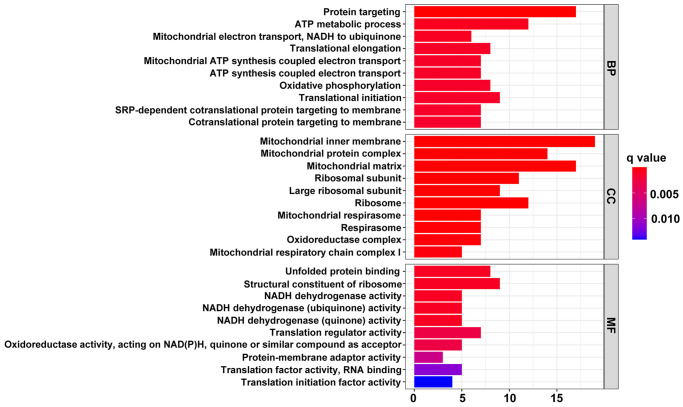
**The top 10 significantly enriched terms in GO enrichment analysis.** The horizontal axis indicates the number of genes enriched in this term. The term color denotes the degree of enrichment.

Functional and pathway enrichment analyses were conducted to investigate the function of the 142 genes mentioned above. The results of Gene Ontology (GO) analysis demonstrated that these highly expressed genes in the HT with LVH group were principally associated with the function and structure of mitochondria, such as the oxidoreductase complex, mitochondrial inner membrane, mitochondrial matrix, ATP metabolic process, mitochondrial electron transport, mitochondrial respirasome, oxidative phosphorylation, and NADH dehydrogenase activity (Figure 4).

**Figure 5:**
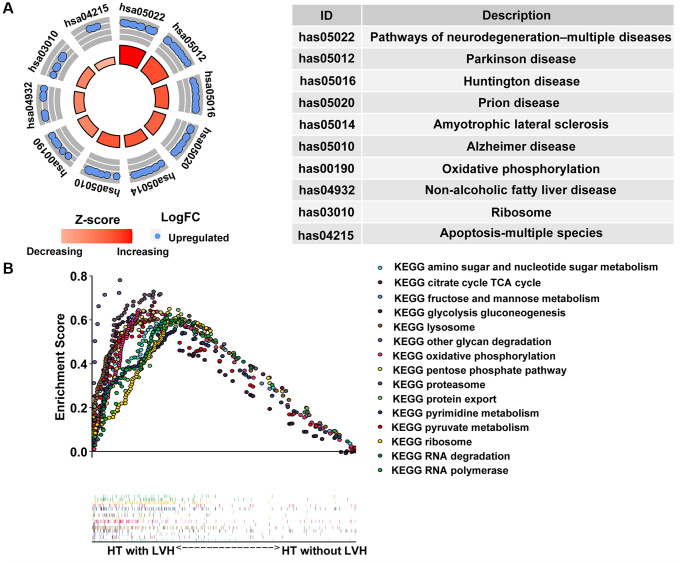
**Pathway enrichment analysis.** (**A**) The circle plot of KEGG enrichment analysis. Each spot in the circle represents a gene, and the outer circle refers to significant enrichment signaling pathways IDs. The inner circle shows the Z-score, the color depth corresponding to the value of the Z-score. The right table annotates the specific KEGG pathways. (**B**) Fifteen significantly enriched pathways in the HT with LVH group through gene set enrichment analysis (GSEA).

The ten most highly enriched pathways in Kyoto Encyclopedia of Genes and Genomes (KEGG) were related to three aspects: (I) mitochondrial abnormalities leading to neuronal degenerative diseases, including Alzheimer's disease (AD), Parkinson's disease (PD), Huntington's disease (HD), amyotrophic lateral sclerosis (ALS), and prion disease; (II) cell apoptosis, like apoptosis-multiple species; (III) other diseases or pathways resulting from dysfunctional mitochondria, such as nonalcoholic fatty liver and oxidative phosphorylation, respectively (Figure 5A). Simultaneously, gene set enrichment analysis (GSEA) was also conducted to uncover the enriched hallmarks and pathways in hypertensive patients with LVH. The results showed that enriched pathways mainly focused on lysosome, proteasome, ribosome, RNA polymerase and degradation, as well as multiple pathways in nutrient metabolism (Figure 5B). Taken together, these results implied that mitochondrial dysfunction in peripheral blood leukocytes might be a potential indicator of HT with LVH.

### Screening for candidate diagnostic biomarker

**Figure 6:**
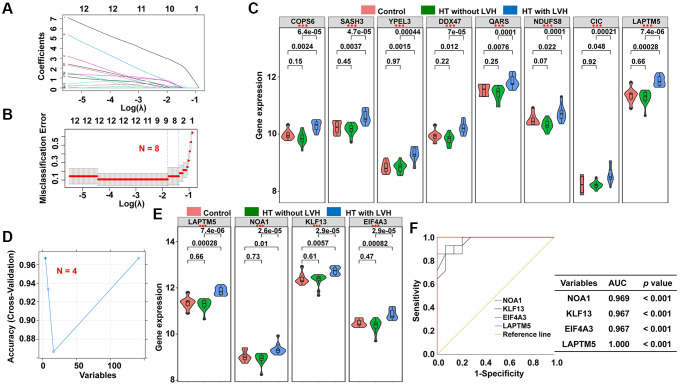
**Screening of potential diagnostic markers for HT with LVH by LASSO and SVM-REF algorithm.** (**A**) LASSO coefficient profiles of the 142 genes. Different color curves signify different genes. The numbers on the top of the figure indicate the number of the candidate genes entering in LASSO regression according to various lambda (λ) values displayed in the bottom of the figure. (**B**) Five-fold cross-validation to select the optimal tuning parameter (λ). The right dashed lines show the optimal values by 1-SE criteria (λ = 0.2729728). (**C**) The difference in expression level of the subset genes among the three groups (^*^p < 0.05; ^**^p < 0.01; ^***^p < 0.001). (**D**) The blue solid points indicate the maximum classification precision through ten-fold cross-validation, and the corresponding genes sets at this point are the best diagnostic markers selected by SVM. (**E**) Violin plots showing the expression patterns of the four genes screened by SVM-RFE algorithm among the three groups (^*^p < 0.05; ^**^p < 0.01; ^***^p < 0.001). (**F**) ROC curve analyses of the four genes filtered by SVM-REF algorithm showing the diagnostic efficacy for HT with LVH subjects.

To discover reliable diagnostic biomarkers to distinguish HT without LVH from HT with LVH, two different algorithms were implemented based on 142 candidate genes discussed previously, the least absolute shrinkage and selection operator (LASSO) binomial regression model and support vector machine-recursive feature elimination (SVM-RFE). According to the plot of LASSO coefficient profiles (Figure 6A) as well as optimal tuning parameter selection map (Figure 6B), λ was set at 0.2729728. Then, eight featured genes with non-zero coefficients were screened out. Meanwhile, risk score in terms of the expression levels of these panel genes and the coefficients obtained by the LASSO regression were calculated for each patient as follows: risk score = (2.52661161 × lysosomal associated transmembrane protein 5 [LAPTM5]) + (0.11600992 × COP9 signalosome subunit 6 [COPS6]) + (0.07153031 × SAM and SH3 domain containing 3 [SASH3]) + (0.18897183 × yippee like 3 [YPEL3]) + (0.31318583 × DEAD-box helicase 47 [DDX47]) + (0.06362767 × glutaminyl-tRNA synthetase [QARS]) + (0.16892870 × NADH ubiquinone oxidoreductase core subunit S8 [NDUFS8]) + (0.30961096 × capicua transcriptional repressor [CIC]). Next, to assess the predictive accuracy to discriminate HT without LVH and hypertensive LVH samples of this risk model, receiver operating characteristic (ROC) curve analysis was performed for the risk score and the individual genes of the signature. The results demonstrated that the area under ROC curve (AUC) values were 1.000, 1.000, 0.946, 0.954, 0.893, 0.944, 0.934, 0.934, and 0.913 for the risk score, LAPTM5, COPS6, SASH3, YPEL3, DDX47, QARS, NDUFS8, and CIC, respectively (Supplementary Figure 2). Moreover, the expression levels of these subset genes were also investigated among the three groups; the results showed that the expression levels of all these genes were significantly higher in hypertensive patients with LVH than in other two groups (Figure 6C). In contrast, there was no significant difference in the expression of these genes between HT without LVH group and Control group (Figure 6C).

Subsequently, a subset of four diagnostic genes were identified using the SVM-RFE algorithm, i.e., LAPTM5, nitric oxide associated 1 (NOA1), Kruppel like factor 13 (KLF13), and eukaryotic translation initiation factor 4A3 (EIF4A3) (Figure 6D). As shown in Figure 6E, the expression levels of these four genes were notably higher in hypertensive LVH samples than in the other two groups. The diagnostic ability of the four potential biomarkers in discriminating HT with LVH from HT without LVH samples displayed a favorable diagnostic value, with an AUC of 0.969 for NOAI, 0.967 for KLF13, 0.967 for EIF4A3, and 1.000 for LAPTM5 (Figure 6F). Ultimately, the gene biomarkers obtained by the two algorithms were overlapped, and one robust diagnostic-related gene, LAPTM5, was obtained for further analysis.

### Correlation analysis of LAPTM5 expression with reactive oxygen species (ROS) and autophagy-related signature to explore the underlying mechanism of LVH

**Figure 7:**
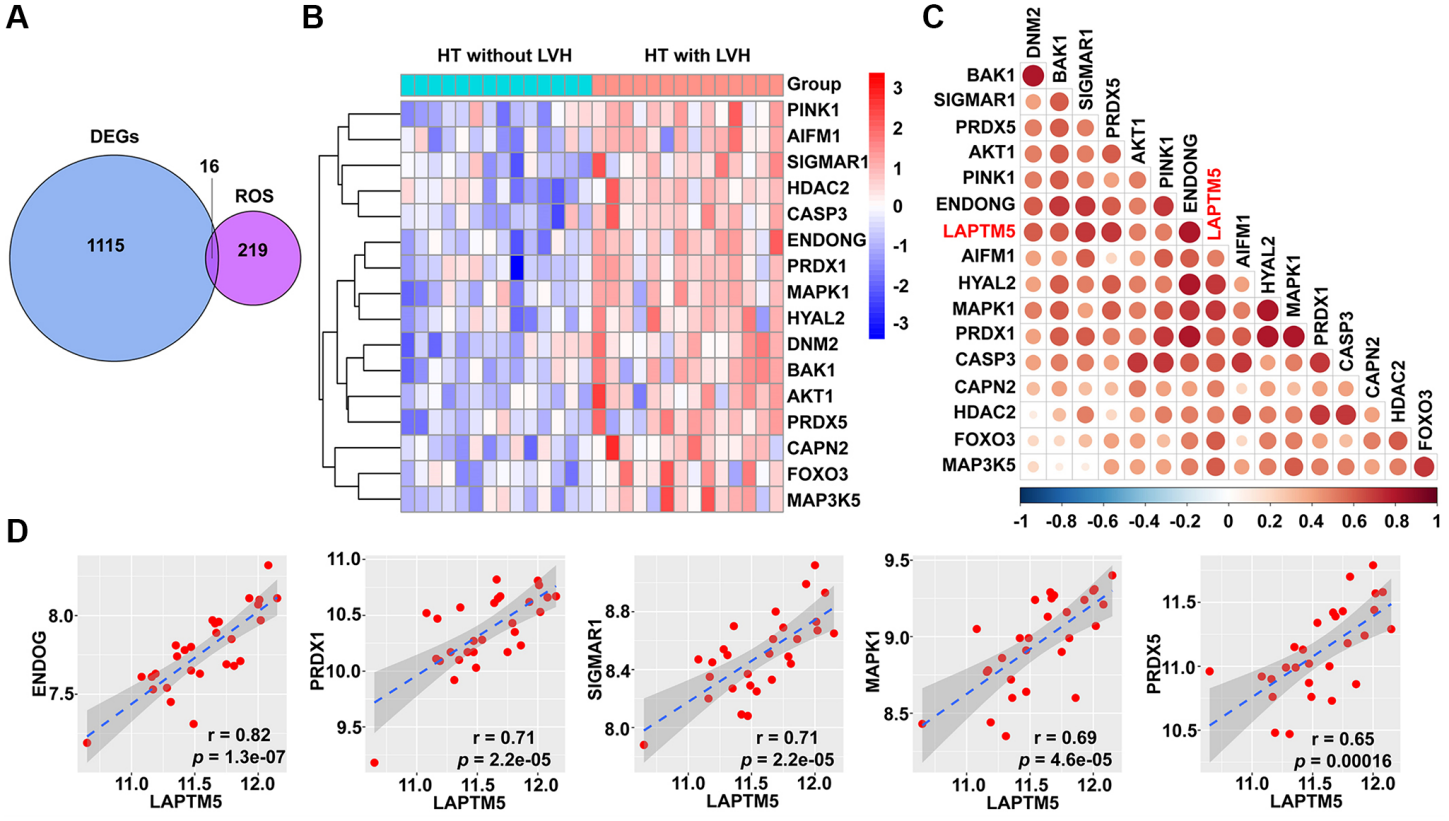
**Correlation between LAPTM5 expression and ROS-related genes in HT with LVH and non-LVH groups.** (**A**) Venn diagram depicts the 16 overlapped DEGs and ROS-related genes. (**B**) Heat map demonstrates the expression profile of differentially expressed ROS-related genes in HT with LVH and without LVH. Genes upregulated (red), downregulated (blue), and unchanged (white) are delineated. (**C**) Heat map of Spearman’s correlation coefficients between LAPTM5 and significantly altered ROS-related genes. The color depth of circle represents the strength of the correlation, red represents a positive correlation, and blue indicates a negative correlation. Darker color indicates stronger correlation. (**D**) Scatterplots show the top five ROS-related genes that have significant positive correlations with LAPTM5. The x-axis shows LAPTM5 expression, and the y-axis shows the expression of ROS-related genes. The Spearman correlation coefficients (r) and corresponding *p* values are shown at the bottom right corner of each plot.

**Figure 8:**
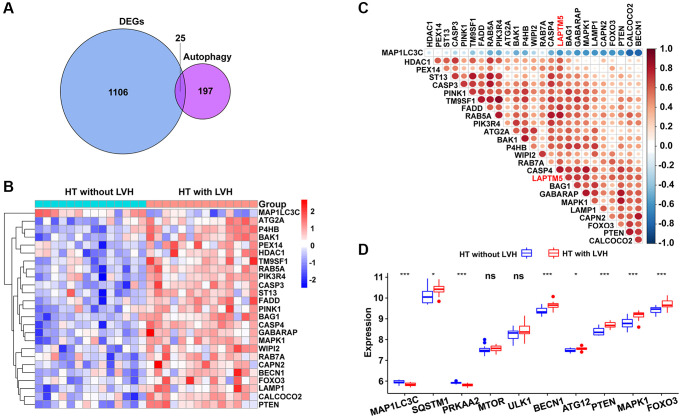
**Association between LAPTM5 expression and autophagy-related genes in HT with LVH and non-LVH groups.** (**A**) Venn diagram showing 25 shared genes by DEGs and autophagy-related genes. (**B**) Heat map illustrates the expression pattern of differentially expressed autophagy-related genes in HT with LVH and without LVH. Red exhibits upregulated genes, while blue stands for downregulated genes in the hypertensive LVH group. (**C**) Spearman’s correlation coefficients heat map showing gene co-expression patterns between LAPTM5 and significantly altered autophagy-related genes. The color depth of circle represents the strength of the correlation, red represents a positive correlation, and blue indicates a negative correlation. Darker color indicates a stronger correlation. (**D**) Box showing the expression levels of 10 autophagy-related genes between HT with LVH and non-LVH groups.

Based on the result of functional and pathway enrichment analysis, compared with HT without LVH subjects, peripheral blood leukocytes of hypertensive patients with LVH displayed mitochondrial dysfunction. Mitochondrial damage leads to the accumulation of ROS, which further induce cell death through autophagy. Therefore, this study focused on investigating the relationship of LAPTM5 expression with ROS- and autophagy-related genes. Sixteen differentially expressed ROS-related genes shown in Figure 7A were found; they were all upregulated in HT with LVH compared with non-LVH groups, as demonstrated in the heat map (Figure 7B). The expression of LAPTM5 presented a significant positive correlation with the majority of significantly altered ROS-related genes (Figure 7C and 7D). Venn diagrams (Figure 8A) showed 25 genes commonly shared by DEGs and autophagy-related genes, and the expression profiles of these distinct autophagy-related genes were visualized on the heat map between HT with LVH and non-LVH samples. It is worth mentioning that most of these genes were overexpressed in hypertensive patients with LVH, except microtubule-associated protein 1 light chain 3 gamma (MAP1LC3C) (Figure 8B). Similar to the results of distribution of expression patterns, a significantly positive correlation was observed between LAPTM5 and changed autophagy-related genes, except MAP1LC3C (Figure 8C). In addition, the expression profiles of 10 key molecules with pivotal roles in autophagic process were compared between the HT with LVH and HT without LVH groups. As shown in Figure 8D, sequestosome 1 (SQSTM1), beclin 1 (BECN1), autophagy related 12 (ATG12), phosphatase and tensin homolog (PTEN), mitogen-activated protein kinase 1 (MAPK1), and forkhead box O3 (FOXO3) were significantly increased in hypertensive LVH samples, while MAP1LC3C and protein kinase AMP-activated catalytic subunit alpha 2 (PRKAA2) were significantly downregulated in hypertensive patients with LVH. The remaining molecules, including mechanistic target of rapamycin kinase (MTOR) and unc-51-like autophagy activating kinase 1 (ULK1), showed no obvious difference between the two groups. Consistently, the scatter plots also exhibited that most of the selected autophagy-related genes significantly positively correlated with the expression level of LAPTM5 (Supplementary Figure 3). Collectively, these results indicated that LAPTM5 may be related to multiple biological processes, such as ROS and autophagy, in orchestrating LVH development.

### LAPTM5-mediated protein–protein interaction (PPI) network construction

**Figure 9:**
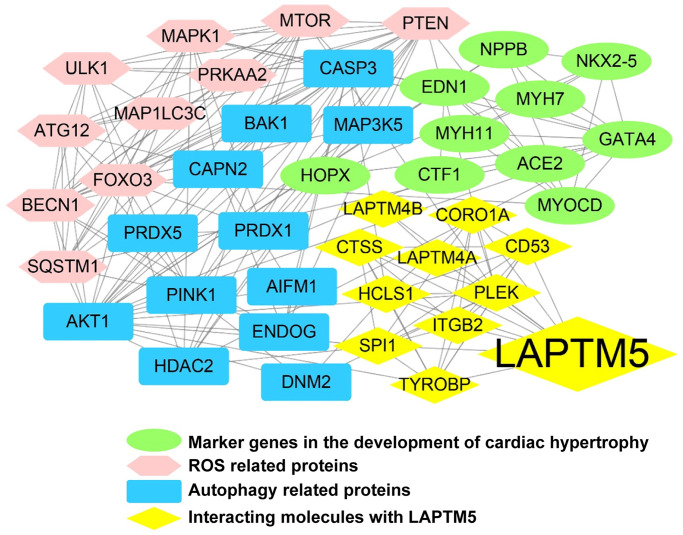
**LAPTM5-mediated PPI network construction.** The node color reflects the source of the proteins. Yellow represents the proteins with strong interactions (score >0.80) with LAPTM5, originating from the STRING database. Brown represents altered ROS-related proteins, blue indicates distinct autophagy-related proteins, and green denotes known key molecules modulated in LVH.

To further elucidate the role of LAPTM5 in hypertensive LVH, the PPI network was constructed. Depending on the source of the proteins mentioned in the methods, the regulatory network consisted of four modules with different colors, including 37 nodes and 150 edges (Figure 9). Thirteen genes, including AKT serine/threonine kinase 1 (AKT1), caspase 3 (CASP3), PTEN, BECN1, FOXO3, MTOR, pleckstrin (PLEK), ATG12, PTEN induced kinase 1 (PINK1), MAPK1, LAPTM5, ULK1, and SQSTM1, were identified with connection degree over 10. As expected, the network demonstrated that LAPTM5 could crosstalk with the other three modules, which possibly indirectly leads to LVH through oxidative stress and cellular autophagy.

### Validation of LAPTM5 expression in peripheral blood leukocytes from mouse model induced by Angiotensin II (Ang II) infusion

**Figure 10:**
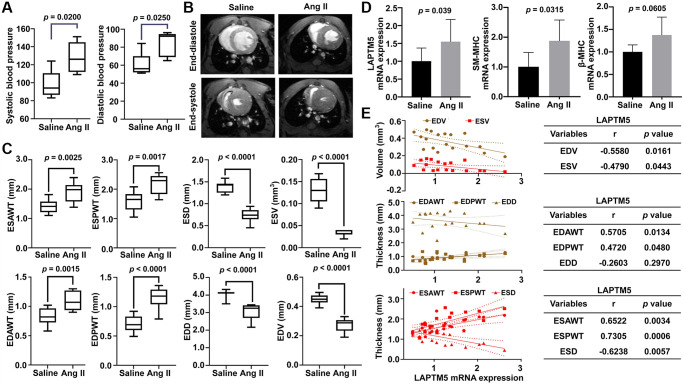
**Assessment of Ang II infusion-induced HT with LVH mouse model.** (**A**) Blood pressure measurement in Ang II-treated mice for 7 days. DBP represents diastolic blood pressure, while SBP indicates systolic blood pressure. (**B**) Representative end-diastolic and end-systolic cine MR images of LV from saline- and Ang II-treated mice for 7 days. (**C**) Quantitative changes in ESAWT, ESPWT, ESD, ESV, EDAWT, EDPWT, EDD, and EDV were compared between the two groups of mice. ESAWT, end-systolic anterior wall thickness; ESPWT, end-systolic posterior wall thickness; ESD, end-systolic diameter; ESV, end-systolic volume; EDAWT, end-diastolic anterior wall thickness; EDPWT, end-diastolic posterior wall thickness; EDD, end-diastolic diameter; EDV, end-diastolic volume. (**D**) RT-PCR analysis of the mRNA expression of LAPTM5, SM-MHC, and β-MHC between the two groups. (**E**) The Pearson correlation analysis between LAPTM5 and cardiac structural parameters of LV. The bottom table shows the correlation coefficients and *p* values. EDV, end-diastolic volume; ESV, end-systolic volume; EDAWT, end-diastolic anterior wall thickness; EDPWT, end-diastolic posterior wall thickness; EDD, end-diastolic diameter; ESAWT, end-systolic anterior wall thickness; ESPWT, end-systolic posterior wall thickness; ESD, end-systolic diameter.

To further confirm whether LAPTM5 is a reliable diagnostic signature in HT with LVH cases, HT with LVH mouse model was developed by Ang II infusion. As shown in Figure 10A, blood pressure was markedly elevated in mice undergoing Ang II infusion for 7 days compared with the saline control groups. CMRI was used to characterize the morphological and functional changes in the left ventricle (LV) between these two groups. In LV short-axis view, Ang II-treated mouse displayed significantly increased thickness of the ventricle wall (Figure 10B), suggesting that mouse model with cardiac hypertrophy was successfully established. Further statistical analysis revealed that the end-systolic anterior wall thickness (ESAWT), end-systolic posterior wall thickness (ESPWT), end-diastolic anterior wall thickness (EDAWT), and end-diastolic posterior wall thickness (EDPWT) were obviously enhanced in the Ang II infusion group, whereas end-systolic diameter (ESD), end-systolic volume (ESV), end-diastolic diameter (EDD), and end-diastolic volume (EDV) were lower compared with the saline control group (Figure 10C). Moreover, mRNA expression of LAPTM5, smooth muscle-myosin heavy chain (SM-MHC), and β-myosin heavy chain (β-MHC) showed an upregulated tendency in leukocytes of Ang II-infused mice relative to the saline controls (Figure 10D). In contrast, there was no statistical difference for other 3 diagnostic genes EIF4A3, KLF13, NOA1 from SVM-RFE algorithm between these two groups (Supplementary Figure 4). In addition, the correlations between the abundance of LAPTM5 and cardiac structural parameters of LV were also examined to further evaluate the diagnostic accuracy and reliability. The results demonstrated that LAPTM5 had a strongly positive connection with ESAWT, ESPWT, EDAWT, and EDPWT, with correlation coefficients between 0.4720 and 0.7305. Conversely, LAPTM5 presented dramatically negative correlation with EDV, ESV, and ESD, with correlation coefficients of −0.558, −0.479, and −0.6238, respectively (Figure 10E).

**Figure 11:**
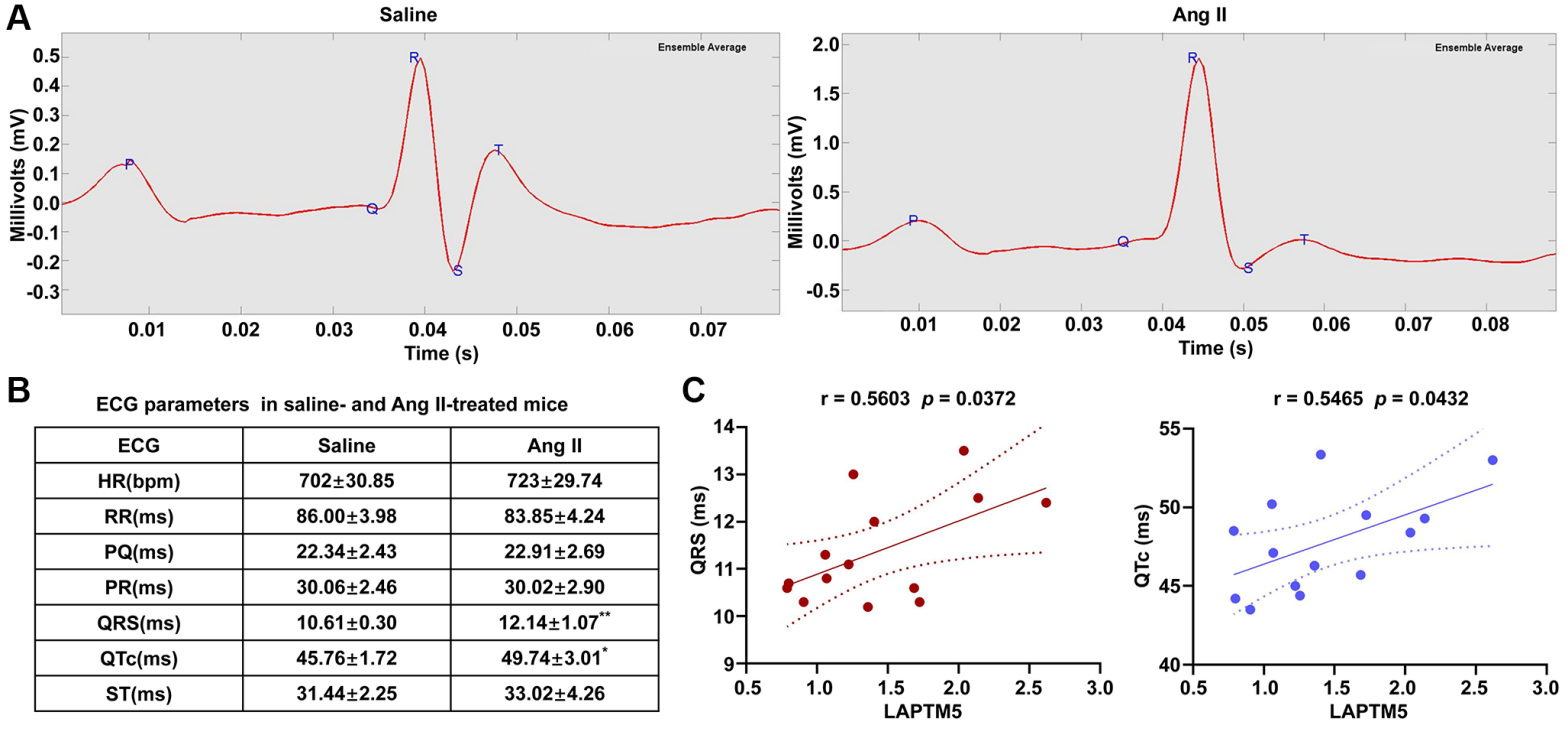
**ECG evaluation in Ang II infusion–induced HT with the LVH mouse model.** (**A**) Representative ECG recordings from saline- and Ang II-treated mice for 7 days. (**B**) Table shows the different of ECG parameters in saline- and Ang II-treated mice for 7 days (^*^p < 0.05, ^**^p< 0.01, ^***^p < 0.001). (**C**) Analysis of Pearson’s correlation between LAPTM5 and the widths of the QRS complex and the QTc interval.

Moreover, ECG analyses were carried out between these two groups to further confirm whether cardiac remodeling had occurred in the Ang II-induced mouse model after 7 days. Compared with saline control groups, the ECG of cardiac hypertrophy in mice is characterized by a wide QRS complex and prolonged QTc interval (Figure 11B), whereas other ECG parameters remained unchanged. Figure 11A shows the representative ECG tracings. Moreover, there was a remarkable positive relation between the mRNA expression level of LAPTM5 and the QRS width, the QTc interval. Correlation coefficients were 0.5603 and 0.5465, respectively (Figure 11C). Taken together, the results confirmed that LAPTM5 could serve as the potential biomarker for hypertensive LVH. Furthermore, hypertension mouse model without cardiac hypertrophy was also developed through Ang II infusion for 1 day. Compared with the saline control group, both systolic pressure and diastolic pressure increased significantly in the Ang II-induced group, while no significant change was observed in other indices concerning left ventricular structure and function through CMRI assessment between these two groups (Supplementary Figure 5A–5C). Correspondingly, the expression abundance of LAPTM5 was also detected by RT-PCR in the hypertension mouse model without cardiac hypertrophy; however, there was no distinct change between the groups (Supplementary Figure 5D). All these findings indicate that LAPTM5 was a potential specific biomarker to designate HT with LVH.

## DISCUSSION

LVH is an adaptation to chronic pressure overload in arterial HT, which allows the LV to maintain volume output against the increased systolic pressure. Hypertrophy is a risk factor for morbidity and mortality in HT, and the presence of LVH worsens the prognosis in hypertensive patients [1–3]. A large proportion of hypertensive patients with LVH are not diagnosed in the early stage of the disease. Moreover, many of them do not undergo timely intervention for early prevention and treatment, which is one of the principal causes of unfavorable patient outcomes. Therefore, identification and investigation of the underlying biomarkers for early-stage screening and diagnosis of cardiac hypertrophy are urgently required. Recently, data mining strategies on public access databases and integrative bioinformatics analysis have been demonstrated to be valid methods to identify potential biomarkers or even new therapeutic targets in complex diseases [13–15]. To the best of our knowledge, the present study was the first to identify candidate diagnostic biomarkers for hypertensive patients with LVH by using the publicly available GEO dataset and comprehensive bioinformatics approaches, which could provide novel insight into the molecular mechanism associated with the pathogenesis of cardiac hypertrophy.

In this study, we extracted transcriptomics data from peripheral blood leukocytes of hypertensive patients with LVH or without LVH and the healthy controls in the GSE74144 dataset through a series of bioinformatics analyses and validated them in subsequent experimental studies. Based on this strategy, we found that most transcriptional similarities between hypertensive patients without LVH and the healthy controls. In this study, we found that there was no difference in the DEGs of leukocytes between control samples and hypertensive patients without LVH. This result was not consistent with the findings of several previous studies [16–19], which may be because the present study is a small-scale, population-based study involving 8 control individuals, 14 HT without LVH, and 14 HT with LVH. Despite novel biomarkers and important molecular targets during the progression of HT using small sample sizes including hypertensive patients and normotensives [20, 21], early diagnosis of HT-related LVH still remains indefinite. To our knowledge, this is the first data-mining study that compares and analyzes the mRNA expression profiles of the peripheral blood cells of healthy individuals and those of hypertensive patients with or without LVH, and uncovers potential biomarkers and therapeutic targets of HT-related LVH. In contrast, the transcriptional profiles of blood leukocytes were markedly differentially expressed in hypertensive patients with LVH compared with the other two groups. To identify the genes most relevant to hypertensive LVH, we performed a WGCNA analysis to select the modules with the strongest correlation between the module traits and genes in the modules. WGCNA is an advanced bioinformatics method for the identification of highly correlated gene modules and hub genes based on a network-focused algorithm [22]. WGCNA can integrate gene expression and trait data effectively to identify functional pathways and candidate biomarkers, which has been extensively utilized in finding the gene–network signature, co-expression modules, and hub genes associated with complicated diseases [23–25]. Here, we combined WGCNA and DEG approaches to explore hub genes highly associated with LVH. We showed 142 DEGs in peripheral blood leukocytes between hypertensive patients with LVH and non-LVH. To further understand the function of these genes, KEGG and GO analyses as well as GSEA enrichment analysis were conducted. The functional enrichment results demonstrated that the DEGs were primarily enriched in oxidative phosphorylation, mitochondrial damage, and degradation functions, such as lysosome and proteasome functions. These findings are in general agreement with the previous finding that mitochondrial respiratory dysfunction of peripheral blood mononuclear cells (PBMCs) may be associated with inflammation and oxidative stress in early-stage heart failure patients [26].

LASSO regression analysis is a shrinkage and variable selection method for linear regression models, which minimizes prediction error for a quantitative response variable by imposing a constraint on the model parameters that cause regression coefficients for some variables to shrink towards zero [27]. SVM-RFE is a machine learning method based on support vector machine, which is used to find the best variables by deleting SVM-generated eigenvectors [28]. The two algorithms are mainly used to screen feature variables and build the best classification model. In this study, LAPTM5 was identified as the diagnostic marker of hypertensive LVH by integrating the union of features from LASSO and SVM-RFE. Furthermore, the ROC curve showed that LAPTM5 might be a potential and promising biomarker for discriminating hypertensive patients with LVH from those without LVH. LAPTM5 is a transmembrane protein that resides in lysosomes and functions as a regulator of protein trafficking [29]. Interestingly, previous studies have shown that LAPTM5 is preferentially expressed in hematopoietic cells and immune cells of lymphoid and myeloid origin, and it acts as a positive regulator of proinflammatory responses by macrophages [30]. We and others have reported that macrophage-mediated inflammation plays a pivotal role in the initiation and progression of hypertensive cardiac remodeling [31–33]. Evidence from previous studies and our own analyses suggests that LAPTM5 may be involved in the development of hypertensive cardiac hypertrophy and have the potential to be used as a diagnostic marker for hypertensive LVH.

It is well established that elevated ROS or oxidative stress induces protein oxidation and dysregulated cell signaling, leading to inflammation, proliferation, apoptosis, migration, and fibrosis, all of which are important processes contributing to impaired vascular function, cardiovascular remodeling, renal dysfunction, immune cell activation, and sympathetic nervous system excitation in HT [34]. Moreover, accumulating evidence suggests that the autophagy–lysosome pathway plays a key role in regulating systematic and local inflammatory responses [35, 36]. It has been reported that LAPTM5-mediated lysosomal destabilization with lysosomal-membrane permeabilization leads to an interruption of autophagic flux, resulting in the accumulation of immature autophagic vacuoles [37]. Since LAPTM5 is essential in the process of digestion of ingested materials in phagocytes and the proteolytic activity of lysosomes, we speculated that LAPTM5 might be associated with autophagy required for ROS production and that it may augment the inflammatory response and cardiac hypertrophy. The data from the present study suggested that signatures of ROS and autophagy were not only significantly upregulated as hypertensive LVH progressed but also showed a strong positive correlation with LAPTM5 expression, indicating their involvement in hypertensive LVH. Then, we constructed a PPI network and identified several hub genes with high connectivity degrees in the PPI network that may serve as important regulators in hypertensive LVH. Our results may help to obtain a deeper understanding of the association between LAPTM5 and ROS as well as autophagy in the development of cardiac hypertrophy. Finally, using a murine Ang II infusion–induced HT model, further validation experiments confirmed that the expression levels of LAPTM5, SM-MHC, and β-MHC in peripheral blood leukocytes from hypertensive mice with LVH significantly increased compared with those from control mice. It has been reported that the elevated mRNA expression levels of SM-MHC and β-MHC can be used as early marker genes in peripheral blood mononuclear cells of patients with hypertrophic cardiomyopathy. Therefore, we used the expression levels of these two genes in the peripheral leukocytes to characterize the hypertensive mouse model with left ventricular hypertrophy [38]. We also observed positive correlation between leukocyte LAPTM5 mRNA level and LV wall thickness. Echocardiography is an important tool to evaluate left ventricular (LV) function in patients with heart disease, and the frontal QRS-T angle has been established as an independent predictor of non-dipper status in hypertensive patients without LVH [39]. In our study, a widened QRS complex and a longer QTc interval were discovered in HT with the LVH mouse model, and the results are similar to those of previous research [40–42]. All these results presented herein were consistent with the trends observed in our bioinformatics analysis.

The following limitations of our study need to be noted. First, this study was a retrospective analysis of the publicly available dataset. Since other clinical information of the patients was not available, we cannot rule out that other factors might have confounded our analysis. Second, the sample size of our study was small, which might have resulted in some deviations in the results. Our results lacked validation by large and prospective clinical studies because we did not have enough blood samples with intact clinical data and without treatment to build the large-scale cohorts. Third, except for bioinformatics analysis, evidence on the molecular mechanisms of LAPTM5 in hypertensive LVH is currently lacking. Finally, as there were no other clinical parameters related to this dataset, we could not confirm that whether or not LAPTM5 is an independent predictor of HT with LVH by multivariate logistic Regression analysis. Therefore, further work should also extend our bioinformatics analyses to *in vivo* and *in vitro* experiments.

Our study is the first to identify LAPTM5 as a potential blood biomarker for predicting LVH in hypertensive patients based on comprehensive bioinformatics analysis combined with the experimental verification strategy. These findings provide novel insights into the pathogenesis of cardiac hypertrophy, which might be helpful for developing a screening biomarker in hypertensive patients to stratify risk of LVH and initiate preventive interventions.

## MATERIALS AND METHODS

### mRNA dataset collection

Microarray gene expression profiles of peripheral blood leukocytes from GSE74144 were obtained from GEO (https://www.ncbi.nlm.nih.gov/geo/) based on the GPL13497 platform, which contained 8 healthy individuals (Control), 14 hypertensive patients without LVH (HT without LVH), and 14 hypertensive patients with LVH (HT with LVH). Then, the probe IDs were mapped to gene symbols and the mean values were computed as the final expression level for these probes with the same gene names in this dataset. Finally, an expression matrix comprising 21,748 gene symbols was acquired.

### Identification of differentially expressed genes (DEGs)

The DEGs between Control and HT without LVH, as well as between HT with LVH and HT without LVH, were screened by R package ‘limma’. A false discovery rate (FDR)-adjusted *p*-value < 0.01 was set as the threshold for DEGs identification. The DEGs screened out were visualized by volcano plot and heat map using R packages ‘gghplot2’ and ‘pheatmap’.

### Construction of weighted gene co-expression network analysis (WGCNA) and identification of modules related to HT with LVH

WGCNA was conducted to identify gene sets (modules) associated with hypertensive LVH using R package ‘WGNCA’. First, the top 5,000 genes with the largest variation between HT without LVH and HT with LVH were selected in the network. Then, two outlier samples were removed for the subsequent analysis through cluster analysis. After that, scale independence and mean connectivity analysis of modules with different power values were carried out to filter the optimal soft threshold for further module analysis. The soft threshold was confirmed when the signed R^2^ value achieved 0.9. Next, correlations among gene expression modules and HT with LVH were explored. The modules with high significance for HT with LVH were selected to execute intramodular analysis, which can identify genes with high significance for HT with LVH as well as high module membership in modules of interest. For each expression profile, gene significance (GS) acted as the absolute value of the associations between individual genes and clinically interesting trait, while module membership (MM) was defined as the correlation between each module eigengene and a given module. Moreover, a scatterplot of gene significance (GS) versus module membership (MM) in selected modules was drawn to demonstrate their correlation. Finally, the DEGs between HT with LVH and HT without LVH were intersected with the genes extracted from the modules with high relevance to HT with LVH, obtaining another set of candidate genes for further analysis. A Venn diagram was plotted to exhibit the overlapped genes using R package ‘VennDiagram’.

### Functional and pathway enrichment analysis

Gene Ontology (GO) and Kyoto Encyclopedia of Genes and Genomes (KEGG) functional enrichment analyses using 142 genes from the last step were conducted with R package ‘clusterProfiler’. Biological processes (BP), molecular function (MF), and cellular components (CC) were covered in the GO enrichment analysis. Only terms with an FDR adjusted *p*-value < 0.01 were deemed statistically enriched. The top 10 enriched terms ordered by an ascending *q* value are shown in the plot.

Gene set enrichment analysis (GSEA) (version 4.1.0) was performed to evaluate the signaling pathways related to HT with LVH. C2.cp.kegg.v6.2. symbols.gmt was chosen as the reference gene set. To determine the enriched pathways, the number of permutations was set at 1000. The normalized enrichment score (NES) and FDR-adjusted *p*-value were measured to indicate significantly enriched gene sets and pathways.

### Construction of the risk assessment model for HT with LVH through LASSO regression

Least absolute shrinkage and selection operator (LASSO) regression was employed to construct a multi-molecule panel signature with 142 genes to discriminate HT without LVH from HT with LVH using ‘glmnet’ package of R software. To acquire more reliable and objective results, five-fold cross-validation was used to identify the optimal lambda value with the minimum misclassification error. Then, the risk score for each subject was obtained using the risk assessment model. Simultaneously, area under the receiver operating characteristic (ROC) curve was utilized to evaluate the diagnostic sensitivity and specificity of the subset genes and risk score, using SPSS25.0 (IBM Corp., Armonk, New York, USA).

### Feature selection by SVM-RFE

Support vector machine-recursive feature elimination (SVM-RFE) is a machine learning method in terms of a support vector machine algorithm, which can effectively extract the informative gene subsets for more reliable classification. Key gene subset was screened out with ten-fold cross-validation through SVM module by R package ‘e1071’. The classification performances of these potential biomarkers between HT without LVH and HT with LVH were evaluated with the area under ROC curve (AUC). Ultimately, potential diagnostic biomarkers were sifted out by overlapping the genes from the LASSO or SVM-RFE model.

### Spearman’s correlation analysis

The relationships between the mRNA expression of the potential diagnostic biomarker and reactive oxygen species (ROS) or autophagy-related genes were investigated using Spearman’s correlation coefficient. mRNA gene sets of ROS (*n* = 285) originated from biological processes (BP) annotated with ‘response to oxidative stress’, the subset of Gene Ontology (GO) downloaded from GSEA (https://www.gsea-msigdb.org/gsea/index.jsp). Autophagy-related genes were derived from the Human Autophagy Database (HADb, http://www.autophagy.lu/), which provided a complete and an up-to-date list of human genes and proteins involved directly or indirectly in autophagy as described in literature. The specific procedures for Spearman’s correlation analysis were as follows: DEGs and ROS or autophagy-related genes were intersected to obtain differentially expressed ROS or autophagy-related genes. Then, the heat maps of Spearman’s correlation coefficients and scatterplots between the expression level of lysosomal associated transmembrane protein 5 (LAPTM5), and ROS or autophagy-related molecules were plotted to clarify the underlying molecular mechanism leading to left ventricular remodeling in hypertensive patients.

### Protein–protein interaction (PPI) network construction

To gain insights into the crucial roles of LAPTM5, a PPI network involving 42 proteins was constructed and analyzed with the online STRING database (https://string-db.org/), followed by reconstruction with Cytoscape software (version 3.8.0, https://cytoscape.org/) after removal of the isolated nodes. These protein molecules were separated into three groups based on their sources: (I) the proteins exhibiting strong interactions (score >0.80) with LAPTM5, generated from the STRING database; (II) DEGs associated with ROS and autophagy, collected from HT with LVH and HT without LVH; (III) molecules associated with hypertensive LVH based on literature review, such as angiotensin II (Ang II), angiotensin-converting enzyme 2 (ACE2), endothelin-1 (EDN1), cardiotrophin 1 (CTF1), natriuretic peptide B (NPPB), homeobox protein Nkx-2.5 (NKX2-5), homeodomain only protein (HOP), transcription factor GATA-4 (GATA4), myosin-7 (MYH7), myocardin (MYOCD), and myosin-11 (MYH11).

### Setup of mouse model using Ang II

Male wild-type C57BL/6J mice were obtained from Beijing Vital River Laboratory Animal Technology Company (Beijing, China) and maintained under specific pathogen-free conditions at the Institute of Materia Medica, Chinese Academy of Medical Sciences, and Peking Union Medical College, China. The mice were separately infused for 1 day, 7 days with saline or a “pressor dose” of Ang II (1,500 ng/kg/min, Sigma-Aldrich) by osmotic mini-pumps (Alzet MODEL 1007D; DURECT, Cupertino, CA, USA) implanted subcutaneously, as previously described [31]. Blood pressure was measured in conscious mice using the tail-cuff method (Visitech Systems, Apex, NC, USA). CMRI and ECG were performed on the mice to assess cardiac hypertrophy development. All of the animal care and experimental protocols complied with the Animal Management Rule of the Ministry of Health, People’s Republic of China (documentation no. 55, 2001) and the Guide for the Care and Use of Laboratory Animals published by the US National Institutes of Health (NIH publication no. 85–23, revised 1996). They were approved by the Institutional Animal Care and Use Committee of Institute of Materia Medica, Chinese Academy of Medical Sciences, and Peking Union Medical College.

### *In vivo* CMRI

CMRI experiments were performed using a PharmaScan 70/16 US (7.0 T, Bruker, Switzerland) MRI scanner after 1 day and then after 7 days of Ang II or saline infusion. A quadrature 1 H resonator was used for radiofrequency transmission (inner diameter = 72 mm) in conjunction with a surface single-loop receive-only coil. First, 2% isoflurane was used to induce anesthesia in the animals, and then the mice were placed in a prone position on dedicated animal beds. We applied an MRI-compatible ECG and respiratory triggering system gating system (PC-SAM, Small Animal Instruments Inc., Stony Brook, NY) to monitor the status of the experimental animal as well as to trigger image acquisition. Anesthesia levels were adjusted as needed in terms of respiratory rate. A stack of contiguous 0.8 mm single slice left ventricular short axis cine-FLASH images were acquired to cover the entire left ventricle (LV). The detailed scan parameters were as follows: repetition time 8.0 ms, echo time 2.4 ms, field of view 25 × 25 mm, matrix size 192 × 192, flip angle 15°. A total of 15 phases images were obtained during one cardiac cycle.

The images were analyzed using ParaVision 6.0.1 (Bruker, Switzerland) to assess the cardiac functional parameters of the LV. LV end-diastolic volume (EDV) and end-systolic volume (ESV) were computed by manually tracing the endocardial border at end-diastole and end-systole images in every sequence, respectively. In addition, LV papillary muscle layer end-diastolic diameter (EDD), end-systolic diameter (ESD), end-diastolic anterior wall thickness (EDAWT), end-diastolic posterior wall thickness (EDPWT), end-systolic anterior wall thickness (ESAWT), and end-systolic posterior wall thickness (ESPWT) were determined.

### Electrocardiography (ECG) recordings in conscious mouse

The ECG of the conscious mice was monitored non-invasively after 7 days of Ang II or saline infusion, using the ECGenie electrocardiography system (Mouse Specifics, Inc., Quincy, MA, USA). ECG signals were acquired with a 6.5 cm × 7 cm footplate electrode, where the mouse’s paws can direct contact of electrodes and move freely. Raw ECG signals were analyzed using e-mouse software (Mouse Specifics). RR, PQ, PR, ST, and HR-Corrected QT (QTc) intervals were calculated; heart rate (HR) and QRS width were also quantified.

### Isolation of peripheral blood leukocytes

After anesthetization, intracardiac blood samples (0.5 mL) were obtained by syringe and placed into tubes containing sodium citrate. Subsequently, the blood samples were incubated with lysis buffer (155 mM NH_4_Cl, 10 mM NaHCO_3_, 5 mM EDTA, pH 7.4) for 5 minutes to remove erythrocytes. After centrifugation, the cell pellets were washed twice with phosphate-buffered saline (PBS) for total RNA extraction.

### Real-time quantitative polymerase chain reaction (RT-PCR)

Total RNA was extracted from blood leukocytes using the RNeasy kit (Beyotime, Shanghai, China, R0027) in line with the manufacturer’s instructions and reverse-transcribed by SuperScript II reverse transcriptase (TaKaRa, Japan, RR047). Real-time PCR was then performed using the SYBR Green Mix (TaKaRa, Japan, RR820) on an ABI 7900 HT Real-Time PCR system. The primer sequences were as follows: LAPTM5, 5′-CGTCTCGTCTCCATCAGCAG-3′, 5′-TGACCCATCCTGTCGTCTGA-3′; smooth muscle-myosin heavy chain (SM-MHC), 5′-GCTCGGGACTCAGACTTCAAT-3′, 5′-GCTGTGGTTGACTCCTGGTG-3′; β-myosin heavy chain (β-MHC), 5′-TGCTGAAGGACACTCAAATCCA-3′, 5′-CCACGATGGCGATGTTCTCTT-3′; EIF4A3, 5′-AATGGGGTTGATATGGACTGTCTTC-3', 5′-TTAGAAAAGATGGCGGAGGCTG-3′ KLF13,5′-CAATAGCTTGGCCTCGTCTC-3′, 5′-AGTGGGTAACACCTGTCAGA-3′ NOA1, 5′-TGAAGGGACTGCTCTGACTG-3′, 5′-GCCACACTTTTCTTCATGCG-3′, GAPDH, 5′-TGGAGAAACCTGCCAAGTATGA-3′, 5′-GGTCCTCAGTGTAGCCCAAG-3′. The relative quantification value of the target, normalized to the housekeeping gene GADPH, was expressed as the fold difference from the mean value of control subjects.

### Statistical analysis

Data analysis and visualization were performed using R software (version 4.0.0) with appropriate packages and GraphPad Prism 8.0 (GraphPad Software Inc., San Diego, CA, USA). For continuous variables, multiple groups were compared by one-way ANOVA, whereas Student’s *t*-test was used for comparisons of two groups. A two-sided *p* < 0.05 was considered to be statistically significant.

### Ethical statement

Experiment protocols were approved by the Institutional Animal Care and Use Committee of Institute of Materia Medica, Chinese Academy of Medical Sciences, and Peking Union Medical College.

## Supplementary Materials

Supplementary Figures

## References

[1] Shenasa M, Shenasa H. Hypertension, left ventricular hypertrophy, and sudden cardiac death. Int J Cardiol. 2017; 237:60–3. 10.1016/j.ijcard.2017.03.002 28285801

[2] Yildiz M, Oktay AA, Stewart MH, Milani RV, Ventura HO, Lavie CJ. Left ventricular hypertrophy and hypertension. Prog Cardiovasc Dis. 2020; 63:10–21. 10.1016/j.pcad.2019.11.009 31759953

[3] Ruilope LM, Schmieder RE. Left ventricular hypertrophy and clinical outcomes in hypertensive patients. Am J Hypertens. 2008; 21:500–8. 10.1038/ajh.2008.16 18437140

[4] Ojji D, Libhaber E, Lamont K, Thienemann F, Sliwa K. Circulating biomarkers in the early detection of hypertensive heart disease: usefulness in the developing world. Cardiovasc Diagn Ther. 2020; 10:296–304. 10.21037/cdt.2019.09.10 32420112 PMC7225418

[5] Seyfeli E, Sarli B, Saglam H, Karatas CY, Ozkan E, Ugurlu M. The Relationship Between High-Sensitivity C-Reactive Protein Levels and Left Ventricular Hypertrophy in Patients With Newly Diagnosed Hypertension. J Clin Hypertens (Greenwich). 2016; 18:679–84. 10.1111/jch.12734 26603359 PMC8031661

[6] Monserrat L, López B, González A, Hermida M, Fernández X, Ortiz M, Barriales-Villa R, Castro-Beiras A, Díez J. Cardiotrophin-1 plasma levels are associated with the severity of hypertrophy in hypertrophic cardiomyopathy. Eur Heart J. 2011; 32:177–83. 10.1093/eurheartj/ehq400 21059734 PMC3021387

[7] Yu X, Xue Y, Bian B, Wu X, Wang Z, Huang J, Huang L, Sun Y. NLR-A Simple Indicator of Inflammation for the Diagnosis of Left Ventricular Hypertrophy in Patients with Hypertension. Int Heart J. 2020; 61:373–9. 10.1536/ihj.19-138 32173694

[8] Kulasingam V, Diamandis EP. Strategies for discovering novel cancer biomarkers through utilization of emerging technologies. Nat Clin Pract Oncol. 2008; 5:588–99. 10.1038/ncponc1187 18695711

[9] Borrebaeck CA. Precision diagnostics: moving towards protein biomarker signatures of clinical utility in cancer. Nat Rev Cancer. 2017; 17:199–204. 10.1038/nrc.2016.153 28154374

[10] Strunz S, Wolkenhauer O, de la Fuente A. Network-Assisted Disease Classification and Biomarker Discovery. Methods Mol Biol. 2016; 1386:353–74. 10.1007/978-1-4939-3283-2_16 26677191

[11] Guo J, Wang Q, Liu Y, Lu L, Hua Y, Hu R, Wang M, Li Z, Wang X, Wang BH, Fu Q, Chen A. Association of expression of ZNF606 gene from monocytes with the risk of coronary artery disease. Clin Biochem. 2018; 60:44–51. 10.1016/j.clinbiochem.2018.08.005 30130524

[12] Li Y, Lin M, Wang K, Zhan Y, Gu W, Gao G, Huang Y, Chen Y, Huang T, Wang J. A module of multifactor-mediated dysfunction guides the molecular typing of coronary heart disease. Mol Genet Genomic Med. 2020; 8:e1415. 10.1002/mgg3.1415 32743916 PMC7549572

[13] Yan Z, Liu L, Jiao L, Wen X, Liu J, Wang N. Bioinformatics Analysis and Identification of Underlying Biomarkers Potentially Linking Allergic Rhinitis and Asthma. Med Sci Monit. 2020; 26:e924934. 10.12659/MSM.924934 32460303 PMC7278529

[14] Ren C, Li M, Du W, Lü J, Zheng Y, Xu H, Quan R. Comprehensive Bioinformatics Analysis Reveals Hub Genes and Inflammation State of Rheumatoid Arthritis. Biomed Res Int. 2020; 2020:6943103. 10.1155/2020/6943103 32802866 PMC7424395

[15] Chen C, Liu GZ, Liao YY, Chu C, Zheng WL, Wang Y, Hu JW, Ma Q, Wang KK, Yan Y, Yuan Y, Mu JJ. Identification of Candidate Biomarkers for Salt Sensitivity of Blood Pressure by Integrated Bioinformatics Analysis. Front Genet. 2020; 11:988. 10.3389/fgene.2020.00988 33101363 PMC7494969

[16] Huan T, Esko T, Peters MJ, Pilling LC, Schramm K, Schurmann C, Chen BH, Liu C, Joehanes R, Johnson AD, Yao C, Ying SX, Courchesne P, et al, and International Consortium for Blood Pressure GWAS (ICBP). A meta-analysis of gene expression signatures of blood pressure and hypertension. PLoS Genet. 2015; 11:e1005035. 10.1371/journal.pgen.1005035 25785607 PMC4365001

[17] Huan T, Meng Q, Saleh MA, Norlander AE, Joehanes R, Zhu J, Chen BH, Zhang B, Johnson AD, Ying S, Courchesne P, Raghavachari N, Wang R, et al, and International Consortium for Blood Pressure GWAS (ICBP). Integrative network analysis reveals molecular mechanisms of blood pressure regulation. Mol Syst Biol. 2015; 11:799. 10.15252/msb.20145399 25882670 PMC4422556

[18] Huang Y, Ollikainen M, Sipilä P, Mustelin L, Wang X, Su S, Huan T, Levy D, Wilson J, Snieder H, Kaprio J, Wang X. Genetic and Environmental Effects on Gene Expression Signatures of Blood Pressure: A Transcriptome-Wide Twin Study. Hypertension. 2018; 71:457–64. 10.1161/HYPERTENSIONAHA.117.10527 29311254 PMC5877117

[19] Zeller T, Schurmann C, Schramm K, Müller C, Kwon S, Wild PS, Teumer A, Herrington D, Schillert A, Iacoviello L, Kratzer A, Jagodzinski A, Karakas M, et al. Transcriptome-Wide Analysis Identifies Novel Associations With Blood Pressure. Hypertension. 2017; 70:743–50. 10.1161/HYPERTENSIONAHA.117.09458 28784648 PMC5997260

[20] Korkor MT, Meng FB, Xing SY, Zhang MC, Guo JR, Zhu XX, Yang P. Microarray analysis of differential gene expression profile in peripheral blood cells of patients with human essential hypertension. Int J Med Sci. 2011; 8:168–79. 10.7150/ijms.8.168 21369372 PMC3047082

[21] Zhao XC, Yang SH, Yan YQ, Zhang X, Zhang L, Jiao B, Jiang S, Yu ZB. Identification of differential gene expression profile from peripheral blood cells of military pilots with hypertension by RNA sequencing analysis. BMC Med Genomics. 2018; 11:59. 10.1186/s12920-018-0378-2 29996846 PMC6042441

[22] Langfelder P, Horvath S. WGCNA: an R package for weighted correlation network analysis. BMC Bioinformatics. 2008; 9:559. 10.1186/1471-2105-9-559 19114008 PMC2631488

[23] Song ZY, Chao F, Zhuo Z, Ma Z, Li W, Chen G. Identification of hub genes in prostate cancer using robust rank aggregation and weighted gene co-expression network analysis. Aging (Albany NY). 2019; 11:4736–56. 10.18632/aging.102087 31306099 PMC6660050

[24] Niu X, Zhang J, Zhang L, Hou Y, Pu S, Chu A, Bai M, Zhang Z. Weighted Gene Co-Expression Network Analysis Identifies Critical Genes in the Development of Heart Failure After Acute Myocardial Infarction. Front Genet. 2019; 10:1214. 10.3389/fgene.2019.01214 31850068 PMC6889910

[25] Feng T, Li K, Zheng P, Wang Y, Lv Y, Shen L, Chen Y, Xue Z, Li B, Jin L, Yao Y. Weighted Gene Coexpression Network Analysis Identified MicroRNA Coexpression Modules and Related Pathways in Type 2 Diabetes Mellitus. Oxid Med Cell Longev. 2019; 2019:9567641. 10.1155/2019/9567641 31915515 PMC6935443

[26] Li P, Wang B, Sun F, Li Y, Li Q, Lang H, Zhao Z, Gao P, Zhao Y, Shang Q, Liu D, Zhu Z. Mitochondrial respiratory dysfunctions of blood mononuclear cells link with cardiac disturbance in patients with early-stage heart failure. Sci Rep. 2015; 5:10229. 10.1038/srep10229 26018291 PMC4448851

[27] Tian GL, Tang ML, Fang HB, Tan M. Efficient methods for estimating constrained parameters with applications to lasso logistic regression. Comput Stat Data Anal. 2008; 52:3528–42. 10.1016/j.csda.2007.11.007 18443660 PMC2352165

[28] Duan KB, Rajapakse JC, Wang H, Azuaje F. Multiple SVM-RFE for gene selection in cancer classification with expression data. IEEE Trans Nanobioscience. 2005; 4:228–34. 10.1109/tnb.2005.853657 16220686

[29] Adra CN, Zhu S, Ko JL, Guillemot JC, Cuervo AM, Kobayashi H, Horiuchi T, Lelias JM, Rowley JD, Lim B. LAPTM5: a novel lysosomal-associated multispanning membrane protein preferentially expressed in hematopoietic cells. Genomics. 1996; 35:328–37. 10.1006/geno.1996.0364 8661146

[30] Glowacka WK, Alberts P, Ouchida R, Wang JY, Rotin D. LAPTM5 protein is a positive regulator of proinflammatory signaling pathways in macrophages. J Biol Chem. 2012; 287:27691–702. 10.1074/jbc.M112.355917 22733818 PMC3431655

[31] Gan W, Ren J, Li T, Lv S, Li C, Liu Z, Yang M. The SGK1 inhibitor EMD638683, prevents Angiotensin II-induced cardiac inflammation and fibrosis by blocking NLRP3 inflammasome activation. Biochim Biophys Acta Mol Basis Dis. 2018; 1864:1–10. 10.1016/j.bbadis.2017.10.001 28986310

[32] McMaster WG, Kirabo A, Madhur MS, Harrison DG. Inflammation, immunity, and hypertensive end-organ damage. Circ Res. 2015; 116:1022–33. 10.1161/CIRCRESAHA.116.303697 25767287 PMC4535695

[33] Jia L, Li Y, Xiao C, Du J. Angiotensin II induces inflammation leading to cardiac remodeling. Front Biosci (Landmark Ed). 2012; 17:221–31. 10.2741/3923 22201740

[34] Touyz RM, Rios FJ, Alves-Lopes R, Neves KB, Camargo LL, Montezano AC. Oxidative Stress: A Unifying Paradigm in Hypertension. Can J Cardiol. 2020; 36:659–70. 10.1016/j.cjca.2020.02.081 32389339 PMC7225748

[35] Levine B, Mizushima N, Virgin HW. Autophagy in immunity and inflammation. Nature. 2011; 469:323–35. 10.1038/nature09782 21248839 PMC3131688

[36] Gukovskaya AS, Gukovsky I, Algül H, Habtezion A. Autophagy, Inflammation, and Immune Dysfunction in the Pathogenesis of Pancreatitis. Gastroenterology. 2017; 153:1212–26. 10.1053/j.gastro.2017.08.071 28918190 PMC6338477

[37] Inoue J, Misawa A, Tanaka Y, Ichinose S, Sugino Y, Hosoi H, Sugimoto T, Imoto I, Inazawa J. Lysosomal-associated protein multispanning transmembrane 5 gene (LAPTM5) is associated with spontaneous regression of neuroblastomas. PLoS One. 2009; 4:e7099. 10.1371/journal.pone.0007099 19787053 PMC2746316

[38] Kontaraki JE, Parthenakis FI, Patrianakos AP, Karalis IK, Vardas PE. Altered expression of early cardiac marker genes in circulating cells of patients with hypertrophic cardiomyopathy. Cardiovasc Pathol. 2007; 16:329–35. 10.1016/j.carpath.2007.04.004 18005871

[39] Tanriverdi Z, Unal B, Eyuboglu M, Bingol Tanriverdi T, Nurdag A, Demirbag R. The importance of frontal QRS-T angle for predicting non-dipper status in hypertensive patients without left ventricular hypertrophy. Clin Exp Hypertens. 2018; 40:318–23. 10.1080/10641963.2017.1377214 28949780

[40] Merentie M, Lipponen JA, Hedman M, Hedman A, Hartikainen J, Huusko J, Lottonen-Raikaslehto L, Parviainen V, Laidinen S, Karjalainen PA, Ylä-Herttuala S. Mouse ECG findings in aging, with conduction system affecting drugs and in cardiac pathologies: Development and validation of ECG analysis algorithm in mice. Physiol Rep. 2015; 3:e12639. 10.14814/phy2.12639 26660552 PMC4760442

[41] Sysa-Shah P, Sørensen LL, Abraham MR, Gabrielson KL. Electrocardiographic Characterization of Cardiac Hypertrophy in Mice that Overexpress the ErbB2 Receptor Tyrosine Kinase. Comp Med. 2015; 65:295–307. 26310459 PMC4549675

[42] Lottonen-Raikaslehto L, Rissanen R, Gurzeler E, Merentie M, Huusko J, Schneider JE, Liimatainen T, Ylä-Herttuala S. Left ventricular remodeling leads to heart failure in mice with cardiac-specific overexpression of VEGF-B_167_: echocardiography and magnetic resonance imaging study. Physiol Rep. 2017; 5:e13096. 10.14814/phy2.13096 28351964 PMC5371547

