# Supplementary material for: Comprehensive bioinformatics analysis identifies LAPTM5 as a potential blood biomarker for hypertensive patients with left ventricular hypertrophy
Source: Aging (Albany NY). 2022 Feb 14;14(3):1508–28. doi: 10.18632/aging.203894 (PMC8876903; doi:10.18632/aging.203894)
Supplement: Supplementary Figures [file aging-14-203894-s001.pdf]

SUPPLEMENTARY FIGURES

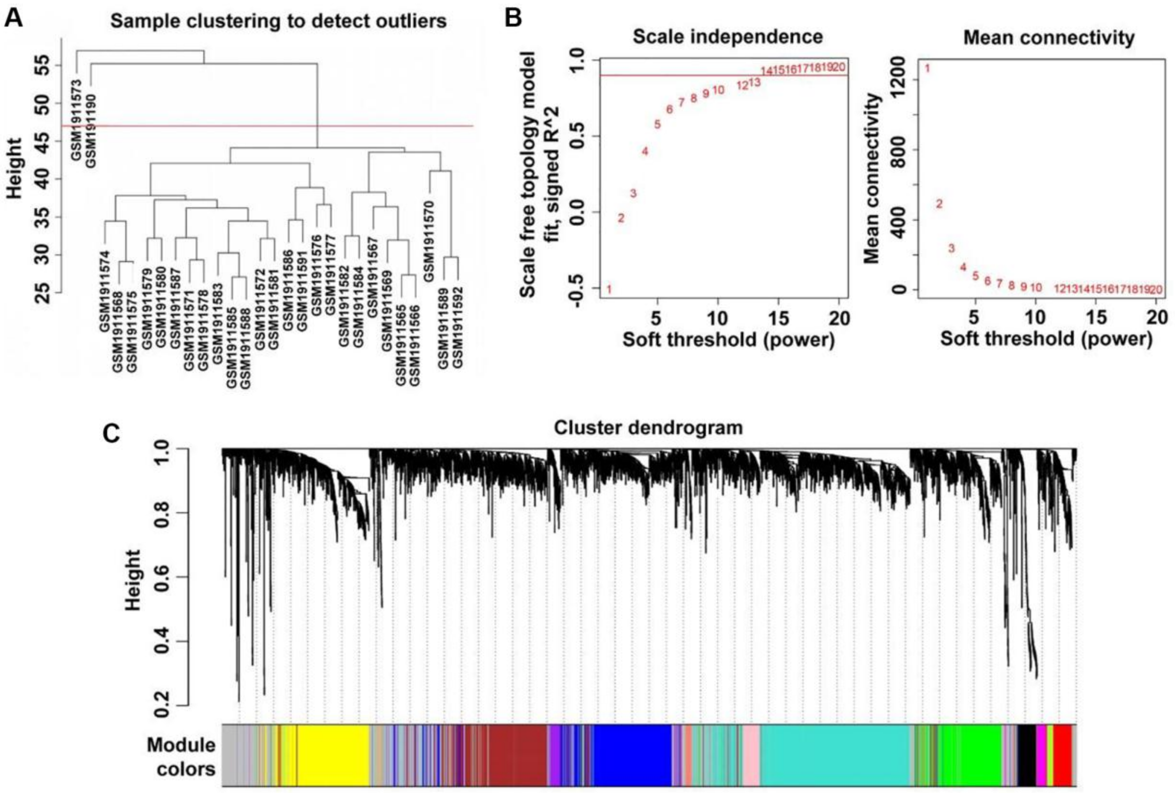

Supplementary Figure 1. Construction of WGCNA co-expression modules. (**A**) Sample clustering was performed to detect outliers. Two deviating samples were excluded because their height surpassed 50. (**B**) Analysis of the scale-free index and mean connectivity of network topology for a set of soft-threshold powers (β). (**C**) Gene clustering tree (dendrogram) obtained based on hierarchical clustering of genes with adjacency-based dissimilarity. The distinctive color band designates the results obtained from the automatic single-block analysis.

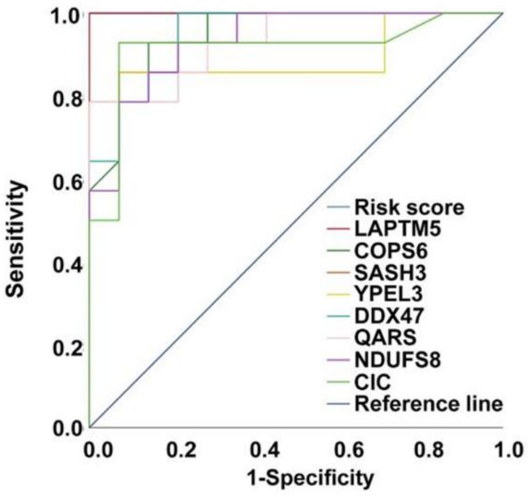

Variables	AUC	*p* value
Risk score	1.000	< 0.001
LAPTM5	1.000	< 0.001
COPS6	0.946	< 0.001
SASH3	0.954	< 0.001
YPEL3	0.893	< 0.001
DDX47	0.944	< 0.001
QARS	0.934	< 0.001
NDUFS8	0.934	< 0.001
CIC	0.913	< 0.001

Supplementary Figure 2. ROC analysis of the eight featured genes originated from LASSO regression for hypertensive patients with LVH and without LVH.

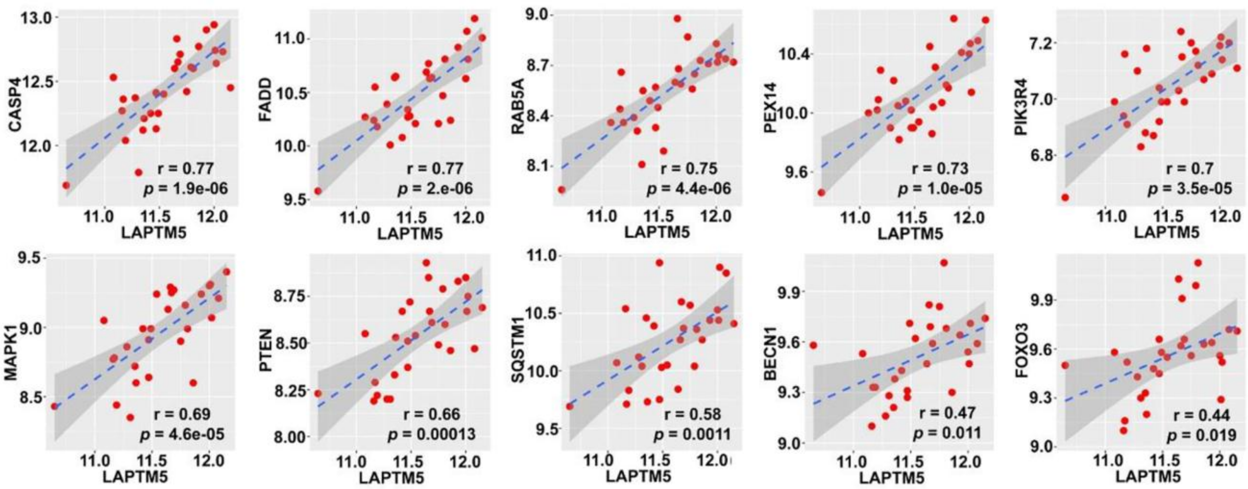

Supplementary Figure 3. Correlations between altered autophagy-related genes and the expression level of LAPTM5 were analyzed by Spearman's correlation coefficients. The x-axis shows LAPTM5 expression, and the y-axis shows the expression of autophagy-related genes. The Spearman correlation coefficients (r) and corresponding *p* values are shown at the bottom right corner of each plot.

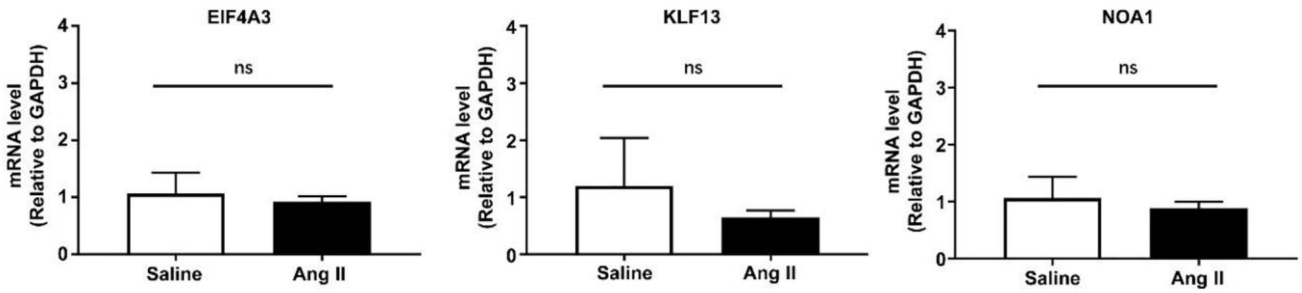

Supplementary Figure 4. RT-PCR analysis of the mRNA expression of EIF4A3, KLF13, and NOA1 between the saline- and Ang II-treated mice for 7 days.

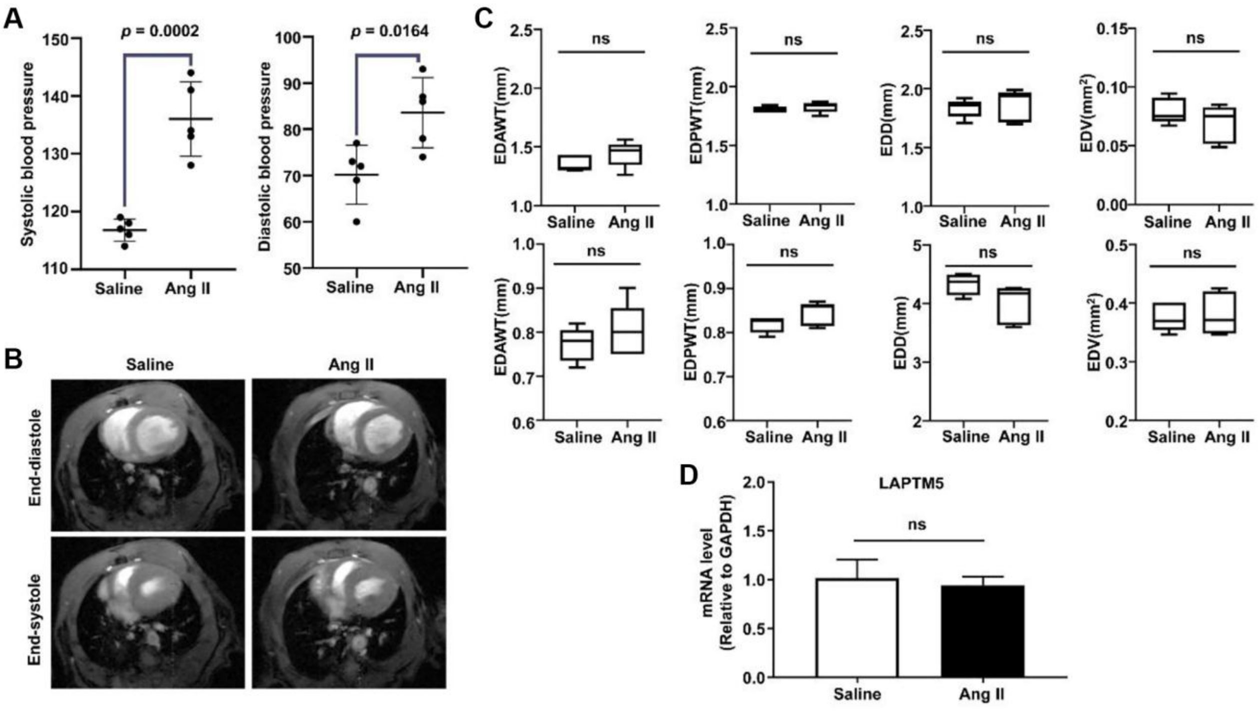

Supplementary Figure 5. Assessment of Ang II infusion–induced HT without the LVH mouse model. (**A**) Blood pressure measurement in Ang II-treated mice for 1 day. DBP represents diastolic blood pressure, while SBP indicates systolic blood pressure. (**B**) Representative end-diastolic and end-systolic cine MR images of LV from saline- and Ang II-treated mice for 1 day. (**C**) Quantitative changes in ESAWT, ESPWT, ESD, ESV, EDAWT, EDPWT, EDD, and EDV were compared between the two groups of mice. (**D**) RT-PCR analysis of the mRNA expression of LAPTM5 between the two groups.
